# Children with craniopharyngioma resection had better response to recombinant human growth hormone therapy than those with idiopathic short stature

**DOI:** 10.3389/fendo.2026.1768846

**Published:** 2026-03-12

**Authors:** Ting Li, Chenyang Li, Xi Wang, Min Nie, Xueyan Wu, Jiangfeng Mao

**Affiliations:** Department of Endocrinology, Peking Union Medical College Hospital, Chinese Academy of Medical Sciences and Peking Union Medical College, Beijing, China

**Keywords:** child, craniopharyngioma, growth hormone, growth hormone therapy, hypopituitarism, idiopathic, recombinant, short stature

## Abstract

**Objective:**

To compare growth response and rhGH dosage requirements between children with craniopharyngioma post-resection (CP group, n=18) and those with idiopathic short stature (ISS group, n=18).

**Methods:**

A retrospective analysis (2010–2020) was conducted on pediatric patients who received rhGH therapy. Key parameters compared between CP and ISS groups included: growth velocity (GV), rhGH dose (IU/kg/d), height, height standard deviation score (HtSDS), IGF-1 standard deviation score (IGF-1 SDS), body mass index (BMI), bone age (BA) and bone age to chronological age ratio (BA/CA).

**Results:**

CP patients were older at baseline with lower HtSDS and BA/CA. Both groups had similar annual growth velocities over three years. The CP group achieved comparable height gains with half the rhGH dose of the ISS group. IGF-1 SDS increased in CP but decreased in ISS, normalizing in both. Long-term, CP patients maintained lower BA/CA and achieved higher terminal height.

**Conclusion:**

In this observational cohort, children with CP received approximately half the rhGH dose used in children with ISS, yet achieved comparable growth velocity and greater near-adult height.

## 1 Introduction

Craniopharyngiomas (CP) are benign tumors originating from the remnants of Rathke’s pouch cells in the sellar region, with a peak incidence during childhood at the age period of 5-14 (1). Clinical presentations include headaches, visual impairments, and hypothalamic-pituitary dysfunction (2, 3). Surgical resection is the primary mode of treatment for CP. Survival rates over a 10-year period among pediatric cohorts ranges from 65% to 100%. However, due to its location near to the hypothalamic pituitary area, surgical interventions may cause catastrophic endocrine dysfunction (1, 4, 5). Most CP patients experience multiple pituitary hormone deficiencies following surgery, necessitating timely initiation of target gland hormone replacement therapy (6, 7).

Growth hormone deficiency (GHD) may profoundly impair linear growth rate and final height, and significantly compromise the long-term quality of life. Supplementation with rhGH can effectively improve growth retardation (8, 9).Based on recent consensus guidelines, GH therapy should be initiated as early as 3 months post-disease remission, with a recommended window of within 1 year, to optimize long-term growth outcomes in pediatric patients (7, 10). Compiling evidence has shown that rhGH therapy in CP is safe and does not augment the risk of tumor recurrence (6, 11, 12).

Idiopathic Short Stature (ISS) refers to individuals whose height falls more than 2.25 standard deviations (SD) below the mean for their corresponding gender and age, without obvious underlying systemic diseases or identifiable endocrine disorders accounting for their growth deficit (14). Approximately 80% of cases of short stature is attributed to ISS. Growth hormone therapy has been utilized to enhance height in ISS since 2003.

In this study, we aim to evaluate the efficacy of rhGH treatment and determine the dosage requirements in patients with ISS and CP. The findings could provide valuable insights into the application of rhGH for addressing growth impairments in both CP and ISS contexts.

## 2 Participants and methods

### 2.1 Participants

**Figure 1:**
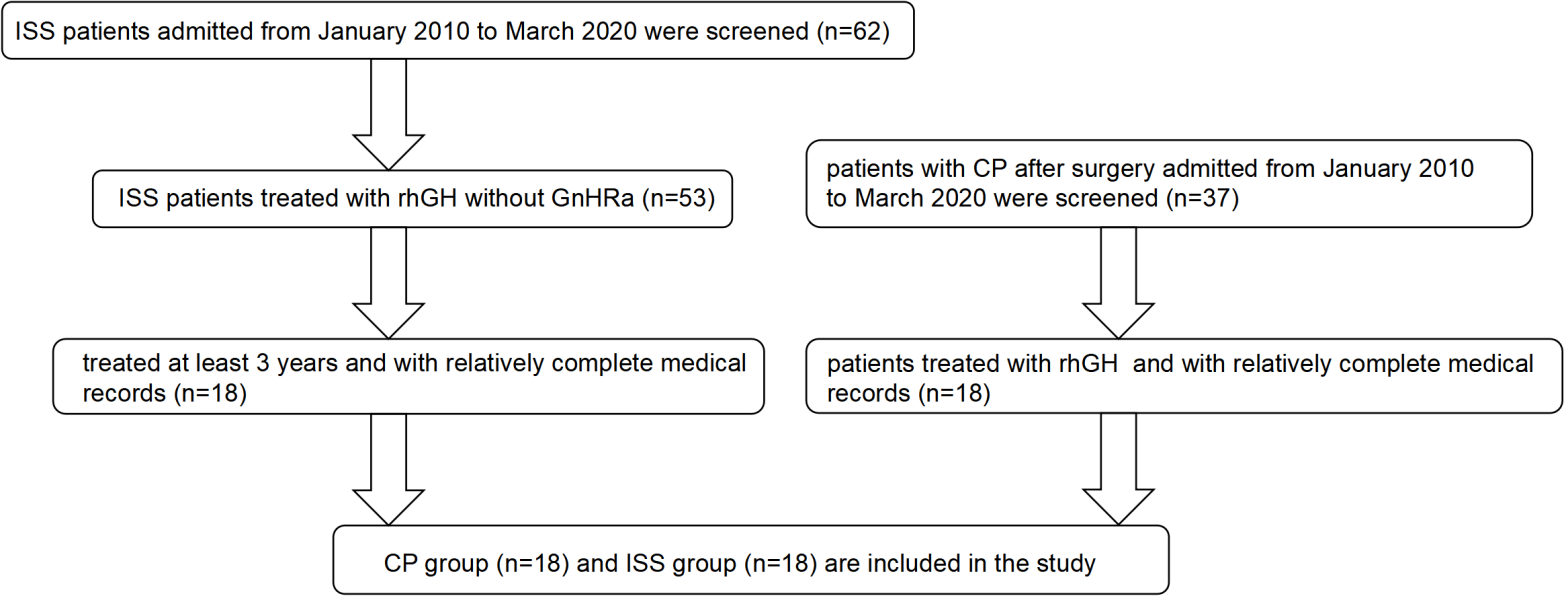
Patient selection flowchart. A total of 62 ISS patients and 37 post-surgical CP patients were screened between January 2010 and March 2020. After excluding those without complete records or insufficient treatment duration, 18 ISS patients and 18 CP patients receiving rhGH therapy were included in the final analysis.

Clinical data was retrospectively collected from outpatients from January 2010 to January 2020. Patients diagnosed with short stature following CP surgery or ISS, and who have been treated with rhGH treated for over three years, were included in this study. The flow chart of patient inclusion can be seen in **Figure 1**.

Diagnostic criteria were as follows: (1) Short stature was defined as a height below -2.0 SD (13) according to growth chart or their GV was significantly slower than expected for their age. Height measurements were standardized by using the same height measurement instrument in our hospital. Three measurements were averaged. (2) CP was confirmed by pathology. (3) ISS was diagnosed after exclusion of systemic, endocrine, chromosomal, or skeletal causes. Genetic testing was not routinely performe, as all patients lacked phenotypic or familial features suggestive of a monogenic disorder. While height ≤ -2.0 SD was used to define short stature, rhGH therapy was initiated only in select patients meeting stricter criteria, including: (i) height ≤ -2.25 SD, or (ii) height between -2.0 and -2.25 SD with growth velocity persistently below the 25th percentile for age, predicted adult height below the normal range, and/or significant psychosocial distress. This approach is consistent with FDA-approved indications that emphasize growth potential and psychosocial factors over absolute height alone (14, 15). (4) Bone age were assessed independently by two physicians, and the average serves as the determination.

Inclusion for CP: For patients with CP, (1) Patient was in a stable condition after surgery without tumor recurrence in repeated MR image at least 1 years; GH therapy was initiated after a postoperative observation period consistent with consensus guidelines (1), when patients demonstrated stable disease remission without tumor recurrence; (2) Growth velocity below 5 cm/year with GHD; (3) They had received rhGH therapy for at least 3 years. Plus, all CP patients had confirmed growth hormone deficiency and were in Tanner stage I (prepubertal) at baseline. Detailed data on other pituitary hormone deficiencies were not available in the retrospective medical records for systematic analysis.

Inclusion for ISS: (1) Fulfill the diagnostic criteria for ISS as outlined above. (2) Met criteria for rhGH initiation per FDA-approved indications and international consensus (14, 15). (3) GH was administered for a minimum duration of 3 years. (4) Patients adhered to prescribed medication and attended scheduled follow-ups. (5) Neither gonadotropin-releasing hormone analogs (GnRHa) nor aromatase inhibitors (AIs) were used in these patients, which may interfere the progression of bone maturation and growth velocity.

Exclusion criteria included: (1) Prior treatment with rhGH or other growth-promoting therapies; (2) Use of GnRHa, aromatase inhibitors, or other agents interfering with bone maturation; (3) Genetic syndromes, chromosomal abnormalities, or chronic systemic diseases affecting growth; (4) Incomplete clinical records or loss to follow-up before 3 years; (5) Prior radiotherapy or chemotherapy for craniopharyngioma (all CP patients in this cohort underwent surgical resection only, without adjuvant radiotherapy); (6) Active tumor recurrence at the time of GH initiation (for CP group).

Patients in the CP (craniopharyngioma) and ISS (idiopathic short stature) groups were matched based on chronological age (± 1 year) and sex at treatment initiation. Due to the retrospective design and limited availability of eligible patients with craniopharyngioma, matching on additional variables such as bone age, BMI, or IGF-1 levels was not performed.

### 2.2 Study design

Parameters including height, weight, BMI, height standard deviation score (HtSDS), growth velocity (GV), IGF-1 SDS, bone age (BA), BA/chronological age ratio (BA/CA), and rhGH dosage were collected at 12, 24, and 36 months of GH therapy and compared between the two groups. For each year, annual GV and rhGH dosage were calculated as the average of all available measurements.

All participants remained prepubertal (Tanner stage I) throughout the 3-year rhGH treatment period. Puberty induction with sex steroids was deliberately avoided to prevent premature epiphyseal fusion and accelerated bone age advancement, which could compromise final height potential—the primary therapeutic goal of this study (16). Consequently, no patient received gonadotropin-releasing hormone analogs (GnRHa), aromatase inhibitors, or exogenous sex steroids during follow-up.

Bone health was monitored clinically; no cases of symptomatic osteopenia or fractures were reported. For patients with confirmed hypogonadotropic hypogonadism (primarily in the CP group), pubertal induction will be considered on an individual basis after completion of the height-focused GH therapy phase, taking into account chronological age, bone age, and psychosocial readiness.

Missing data arose from either (1) patient discontinuation or loss to follow-up, or (2) clinical discretion not to perform specific tests (e.g., IGF-1 or bone age assessment) during routine visits. No imputation was performed; all analyses were based on observed data only. In addition, near-adult height, defined as the patient’s standing height at the last clinical follow-up visit, and the corresponding HtSDS at near-adult height were recorded as key outcome measures.

### 2.3 Statistical methods

Prism software was used for statistical processing. Normally distributed data were presented as mean ± standard deviation (x ± s), while non-normally distributed data were presented as median [Q1, Q3]. Baseline comparisons between groups were made using independent sample t-tests if data met normality and homogeneity of variances; otherwise, Mann-Whitney U tests were used. Intra-group changes after treatment were assessed using paired-sample t-tests if differences were normally distributed; otherwise, Wilcoxon signed-rank tests were used. Normality was tested using the Shapiro-Wilk test, and homogeneity of variances was tested using Levene’s test.

Due to baseline imbalances in age and initial height between the two groups, subsequent inter-group comparisons of outcomes (e.g., changes in height SDS, final height SDS) were performed using analysis of covariance (ANCOVA), with age and initial height as covariates, to adjust for these potential confounding factors. The reported P values for these comparisons are derived from the ANCOVA models. Statistical significance was set at P < 0.05.

## 3 Results

### 3.1 Baseline characteristics of the participants in the CP and ISS groups

There are 18 and 18 patients in CP (M/F=12/6) and ISS (M/F=8/10) group respectively. The mean age at baseline was 10.8 ± 2.97 and 8.5 ± 2.27 years for the CP and ISS group, respectively (P = 0.016). And the baseline BA in two groups was 8.14 ± 3.14 and 8.07 ± 2.45 (n=17) (P = 0.939). Baseline BA/CA in two groups was 0.741 ± 0.152 and 0.91[0.76, 1.04] (n=17) (P = 0.009). Baseline height in two groups was 134 ± 19.53 and 125.5 ± 12.61 cm (P = 0.133), respectively. Height SDS was -1.94 ± 1.66 and -1.57 ± 0.66, respectively (P = 0.727). More detailed information is provided in **Table 1**. All 18 CP patients were in Tanner stage I (prepubertal) at baseline with confirmed GHD. Given the study’s focus on growth velocity and rhGH dosing, analysis of other endocrine axes was not performed. Despite efforts to match participants by sex and target age range, the CP group was significantly older in chronological age and had higher body weight and BMI, but exhibited a significantly lower BA/CA ratio compared to the ISS group (all P < 0.05).

**Table 1:** Baseline characteristics of the participants in two groups.

Items	CP group (n=18)	ISS group (n=18)	P value
Boys/Girls	12/6	8/10	
Baseline			
Age (y)	10.8 ± 2.97	8.5 ± 2.27	0.016
Height (cm)	134 ± 19.53	125.5 ± 12.61	0.133
HtSDS	-1.94 ± 1.66	-1.57 ± 0.66	0.727
Weight (kg)	36.22 ± 13.62(n=16)	25.44 ± 6.11(n=17)	0.006
BMI (kg/m^2^)	18.68 ± 4.12(n=16)	15.66 ± 1.58(n=17)	0.013
Bone age (y)	8.14 ± 3.14	8.07 ± 2.45 (n=17)	0.939
BA/CA	0.741 ± 0.152	0.91[0.76, 1.04] (n=17)	0.009
IGF-1 SDS	-1.93 ± 3.33 (n=17)	1.65 ± 2 (n=16)	0.001
Tanner stage	n=15	n=11	0.999
Tanner I	15	11	
Tanner II	0	0	
Tanner III	0	0	
Tanner IV	0	0	
After three years of GH therapy			
Height (cm)	146.5 ± 12 (n=11)	143.2 ± 6.58 (n=9)	0.477
HtSDS	-0.73 ± 1.09 (n=11)	-0.64 ± 0.85 (n=9)	0.845
Weight (kg)	39.73 ± 9.59 (n=11)	34.06 ± 5.66 (n=9)	0.136
BMI (kg/m^2^)	18.29 ± 2.87 (n=11)	16.49 ± 1.53 (n=9)	0.106
Bone age (y)	9.13 ± 3.1 (n=8)	10.93 ± 2.01 (n=7)	0.212
BA/CA	0.71 ± 0.18 (n=8)	0.97 ± 0.08 (n=7)	0.004
IGF-1 SDS	-0.09 ± 1.61 (n=11)	0.50 ± 1.23 (n=9)	0.374
Near-adult Height	168.2 ± 8.77 (n=15)	154.4[149.7, 158.5] (n=14)	0.029
Near-adult HtSDS	0.4 ± 0.63 (n=11)	-0.32 ± 0.80 (n=14)	0.023
Δ total-HtSDS	1.77 ± 1.33 (n=11)	1.32 ± 0.85 (n=14)	0.062

a. Data are presented as mean ± standard deviation for normally distributed variables and median [Q1, Q3] for non-normally distributed variables.

b. All values are based on non-missing data unless otherwise indicated.

c. CP, craniopharyngioma; ISS, idiopathic short stature; BA, bone age; BA/CA, bone age/chronological age ratio; BMI, body mass index; CA, chronological age; GH, growth hormone; HtSDS, height standard deviation score; IGF-1, insulin-like growth factor 1; SDS, standard deviation score.

### 3.2 Higher final HtSDS in the CP group compared to the ISS group

**Figure 2:**
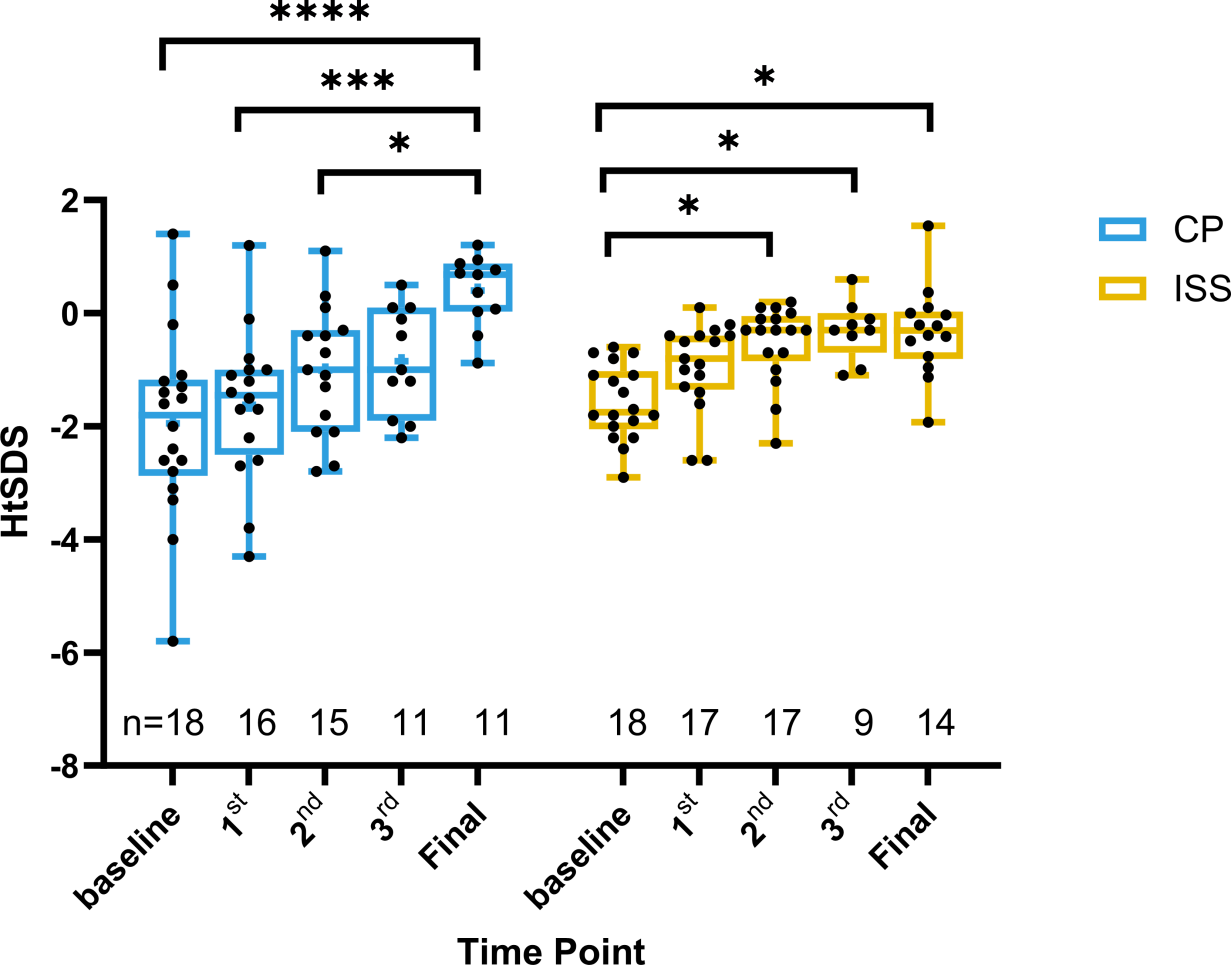
Changes in height standard deviation score (HtSDS) over time in CP and ISS groups. Box plots show HtSDS at baseline, 1^st^, 2^nd^, and 3^rd^ year, and final visit for the CP (blue) and ISS (yellow) groups. Individual data points are shown. Significant differences within group changes over time are indicated by *P < 0.05, ***P < 0.001, ****P < 0.0001. All values represent unadjusted observed data; sample sizes are shown below each time point.

**Figure 3:**
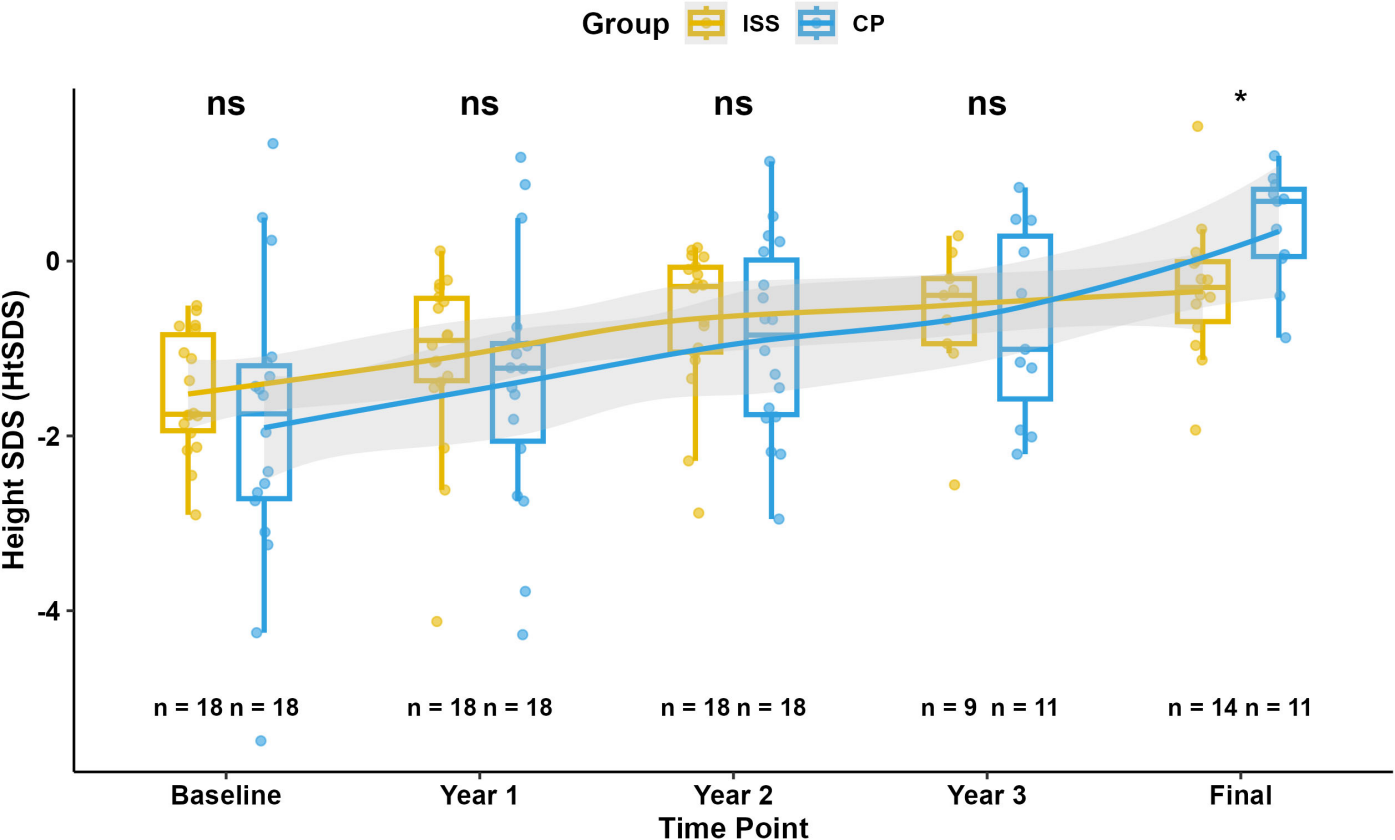
Longitudinal comparison of HtSDS between CP and ISS groups.Box plots show HtSDS at baseline, Year 1, Year 2, Year 3, and final visit for the ISS (yellow) and CP (blue) groups. Individual data points are shown. Solid lines represent median trends with shaded areas indicating interquartile ranges. Group comparisons at each time point are indicated by *P < 0.05 or ns (Mann-Whitney U test on unadjusted data). Sample sizes are shown below each group.

The mean height was 146.5 ± 12 (n=11) and 143.2 ± 6.58 (n=9) cm for the CP and ISS group, respectively (P = 0.477) after 3 year of hormone treatment. Their weight was 39.73 ± 9.59 (n=11) and 34.06 ± 5.66 kg (n=9) (P = 0.136), respectively. Their HtSDS improved to -0.73 ± 1.09 (n=11) and -0.64 ± 0.85 (n=9), (P = 0.845), respectively. Additionally, the final HtSDS of patients in CP group [0.4 ± 0.63 (n=11)] were significantly higher than those in ISS group [-0.32 ± 0.80 (n=14)], (p=0.023) More detailed information is provided in **Figure 2** and **Figure 3**.

### 3.3 Similar growth velocity with lower rhGH dosage in the CP Group compared to the ISS group

**Figure 4:**
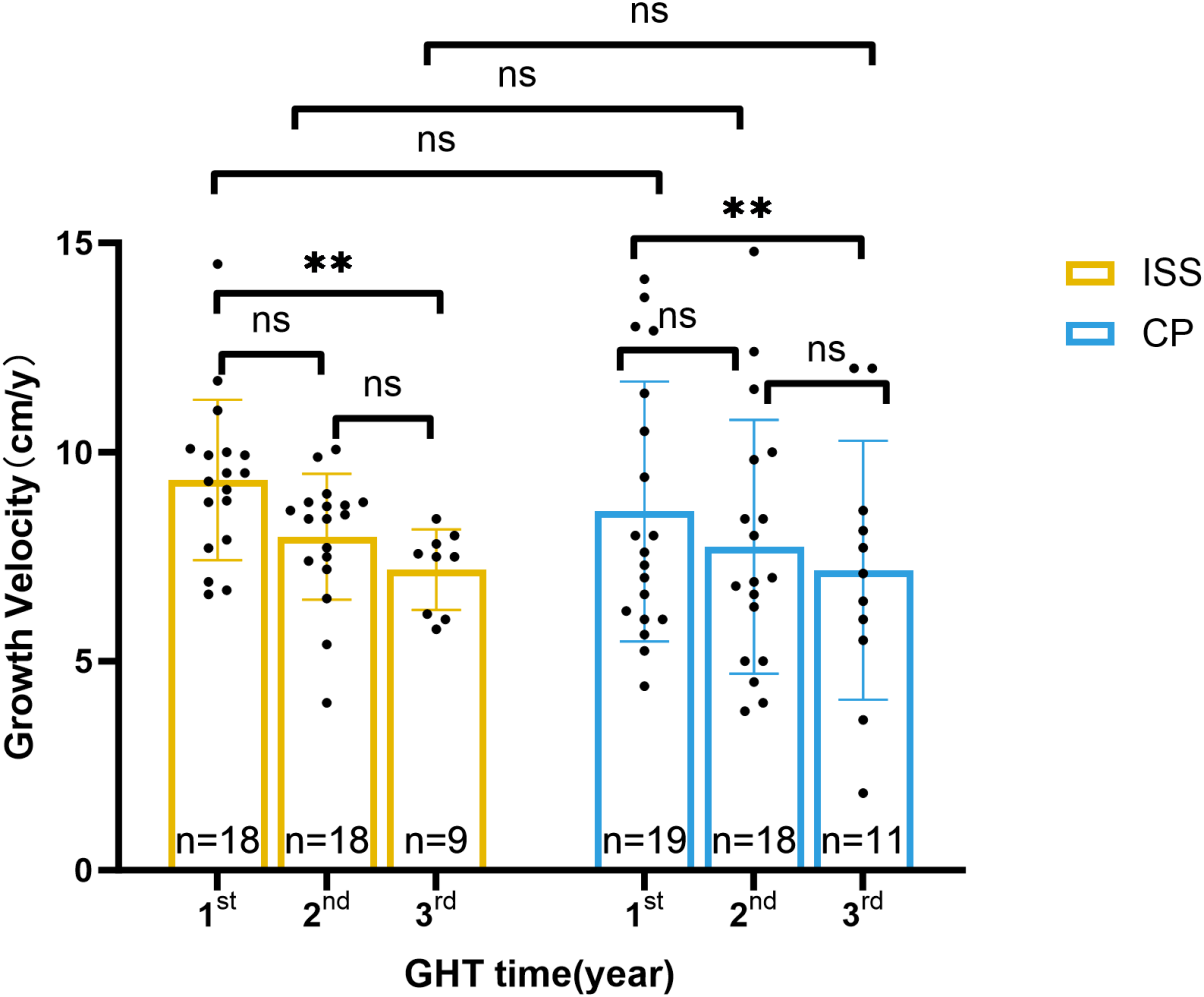
Growth velocity during rhGH treatment in ISS and CP groups. Bar plots show annual growth velocity (cm/year) for the ISS (yellow) and CP (blue) groups at the 1^st^, 2^nd^, and 3^rd^ year of treatment. Individual data points are shown. Significant differences between groups or within-group changes over time are indicated by **P < 0.01, or ns (Mann-Whitney U test for between-group comparisons; Wilcoxon signed-rank test for within-group changes). Statistical tests were applied to raw,unadjusted measurements.Sample sizes are shown below each bar.

**Figure 5:**
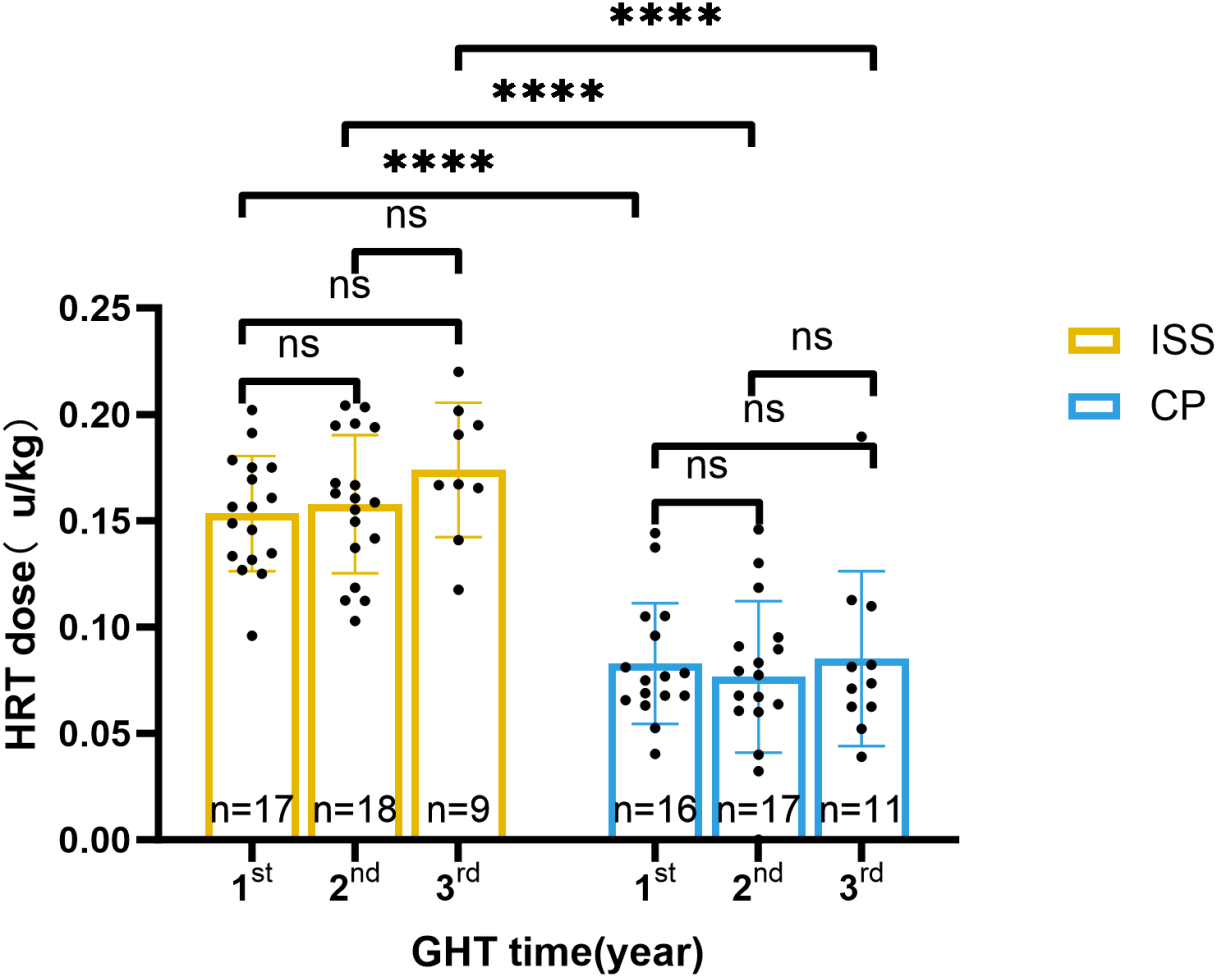
Annual HRT dose during rhGH treatment in ISS and CP groups. Bar plots show the annual HRT dose (mg/kg/d) for the ISS (yellow) and CP (blue) groups at the 1^st^, 2^nd^, and 3^rd^ year of treatment. Individual data points are shown. Significant differences between groups or within-group changes over timeare indicated by ****P < 0.0001, or ns (Mann-Whitney U test for between-group comparisons; Wilcoxon signed-rank test for within-group changes). Statistical tests were applied to raw, unadjusted measurements.Sample sizes are shown below each bar.

After adjusting the baseline age and height, the GV in CP group in the 1^st^, 2^nd^, 3^rd^ year was 7.45[6, 11.78], 7.9 ± 3.05 (n=17), and 7.17 ± 3.09 (n=11) cm per year (P = 0.2438), respectively. Dosage of rhGH was 0.0805 ± 0.0321 (n=15), 0.0762 ± 0.0260 (n=17), and 0.0748 ± 0.0247 (n=11) IU/kg/d (P = 0.8661), accordingly. In ISS group, the GV in the 1^st^, 2^nd^, 3^rd^ year was 9.33 ± 1.92, 8.45[7.35, 8.8], and 7.18 ± 0.96 (n=9) cm per year (P = 0.0042), respectively. Dosage of rhGH was 0.1624 ± 0.0221 (n=17), 0.1672 ± 0.0276, and 0.1739 ± 0.0317 (n=9) IU/kg/d in each year (P = 0.0961). A comparative analysis between two groups across the first to third years of treatment showed that similar growth velocity was found (all P >0.05), while rhGH dosage in CP group was about half of that in ISS group (all P < 0.0001). More detailed information is provided in **Table 2** and **Figures 4**, **5**.

**Table 2:** Growth velocity and GH dosage in CP and ISS groups.

Items	CP group (n=18)	ISS group (n=18)	P value
Boys/Girls	12/6	8/10	
First year of therapy			
GV (cm/y)	7.45 [6, 11.78]	9.33 ± 1.92	0.910
Growth hormone dose (IU/kg/d)	0.0805 ± 0.0321 (n=15)	0.1624 ± 0.0221 (n=17)	<0.0001
Second year of therapy			
GV (cm/y)	7.9 ± 3.05 (n=17)	8.45 [7.35, 8.8]	0.4287
Growth hormone dose (IU/kg/d)	0.0762 ± 0.0260 (n=17)	0.1672 ± 0.0276	<0.0001
Third year of therapy			
GV (cm/y)	7.17 ± 3.09 (n=11)	7.18 ± 0.96 (n=9)	0.9988
Growth hormone dose (IU/kg/d)	0.0748 ± 0.0247 (n=11)	0.1739 ± 0.0317 (n=9)	<0.0001

a. Data are presented as mean ± standard deviation for normally distributed variables and median [Q1, Q3] for non-normally distributed variables.

b. All values are based on non-missing data unless otherwise indicated.

c. CP, craniopharyngioma; ISS, idiopathic short stature; GV, growth velocity.

### 3.4 Persistently lower BA/CA ratio in the CP group compared to the ISS group

**Figure 6:**
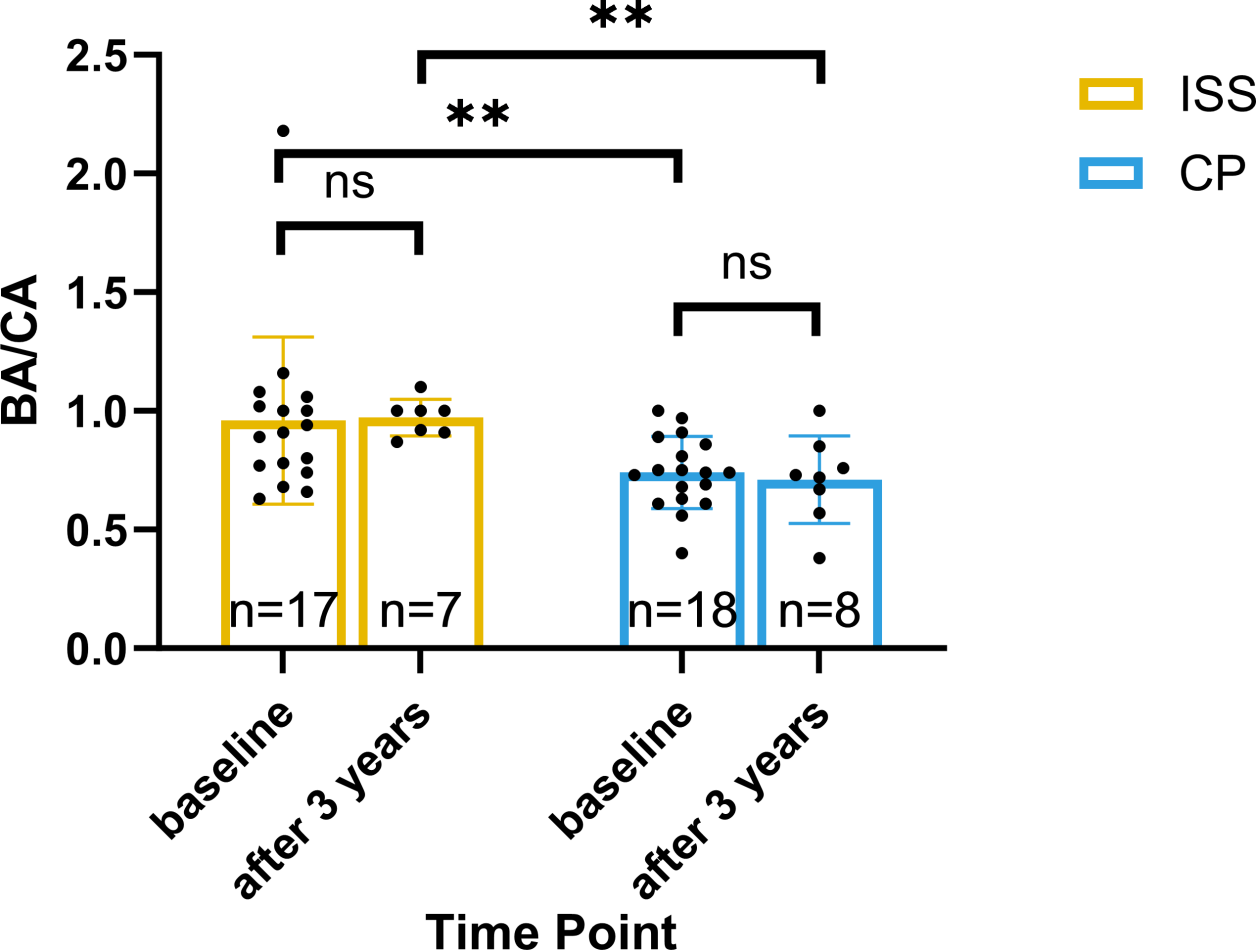
BA/CA ratio at baseline and after 3 years of rhGH treatment. Bar plots show BA/CA ratios for the ISS (yellow) and CP (blue) groups at baseline and after 3 years of treatment. Individual data points are shown. Significant differences between groups or within-group changes over time are indicated by **P < 0.01, or ns (Mann-Whitney U test for between-group comparisons; Wilcoxon signed-rank test for within-group changes). Statistical tests were applied to raw, unadjusted measurements.Sample sizes are shown below each bar. Sample sizes are shown below each bar.

At baseline, the BA/CA in CP and ISS were 0.741 ± 0.152 and 0.91[0.76, 1.04] (n=17), respectively (P = 0.009). Following three years of treatment, BA/CA were 0.71 ± 0.18 (n=8) and 0.97 ± 0.08 (n=7), respectively (P = 0.004). From the baseline, the BA/CA of CP group was statistically lower than that of ISS group, and it was still lower than that of ISS group after treatment, indicating that the immaturity of bone age in CP group, suggesting greater growth potential. More detailed information is provided in **Figure 6**.

### 3.5 Lower baseline IGF-1 SDS but higher final height in the CP group compared to the ISS group

At baseline, the Serum IGF-1 SDS in CP and ISS groups were -1.93 ± 3.33 (n=17) and 1.65 ± 2 (n=16), respectively (P = 0.001). Following three years of treatment, Serum IGF-1 SDS were -0.09 ± 1.61 (n=11) and 0.50 ± 1.23 (n=9), respectively (P = 0.374). After adjusting the baseline age and height, the final height of the CP and ISS groups were 168.2 ± 8.77 (n=15) and 154.4[149.7, 158.5] (n=14), respectively (P = 0.029).

### 3.6 Safety profile

No significant adverse events were reported during rhGH therapy in this cohort. Importantly, no tumor recurrence was observed in the CP group during the study’s follow-up period.

## 4 Discussion

Our investigation showed that children with CP maintained growth rates during rhGH therapy, with annual growth velocities comparable to those in the ISS group. Notably, the CP group achieved similar growth outcomes despite receiving approximately half the rhGH dose administered to ISS patients and having lower baseline IGF-1 SDS. There are many other similar studies, but ours is the first to suggest such a law. In Zheng’s study (9), 0.1 IU/kg/d dosage of rhGH was administered to six children with CP after surgery, exceeding the CP group in our research, yet yielding an improved growth rate of approximately 12 cm/year. Note that Zhang’s cohort had a relatively younger baseline age (10.2 ± 4.2 years) and comprised only six cases. Maghnie’s (6) investigation on the efficacy and safety of rhGH in children drew from a substantial KIGS cohort of 55,284, where the initial rhGH dose varied by diagnosis. For craniopharyngioma, the dose was 0.0729 IU/kg/d (range: 0.0343-0.1114 IU/kg/d), leading to a height standard deviation score improvement from -2.25 (-4.13 to -0.40) pre-treatment to -1.51 (-3.44 to 0.34) after one year of therapy. As far as safety was concerned, no CP patients experienced tumor recurrence during the treatment. This outcome is particularly reassuring, underscoring the safety of GH therapy in this patient population. Additionally, no significant side effects were reported either in the CP group or in the ISS group.

Both groups showed improvements in HtSDS over time. Skeletal maturation, assessed by BA/CA ratio (17, 18), remained stable during follow-up in both cohorts. Notably, the BA/CA ratio was consistently lower in the CP group compared to the ISS group throughout treatment, indicating a relatively delayed skeletal maturation trajectory, which may be associated with a prolonged growth window and greater potential for height gain. The observed growth pattern—favorable response despite lower IGF-1 SDS and reduced rhGH dosing—supports the possibility of enhanced rhGH sensitivity in CP patients (19, 20). However, the underlying mechanisms require further investigation in larger prospective studies.

The two groups were not comparable in several parameters at baseline. (1) The CP group exhibited an older average age at the baseline. It was because they would wait for 1–2 year observational period prior to starting GH therapy in case of tumor recurrence (21, 22). However, there was no statistical difference in BA between the two groups, indicating comparable skeletal maturity and thus similar growth potential at treatment initiation (23). (2) CP group had a similar baseline height compared to ISS group despite older chronological age, possibly due to similar bone age and neither GnRHa nor AIs were used in these patients, which may interfere the progression of bone maturation and growth velocity (16). (3) CP group displayed a higher baseline weight, which may linked to hypothalamic obesity, a common complication associated with CP. Patients with CP usually have disturbances in appetite regulation and energy metabolism, often leading to excessive weight accumulation (24).

Growth hormone therapy promotes linear growth in patients with CP, particularly in the context of underlying growth hormone deficiency. A gradual decline in annual growth velocity was observed over the treatment period, which is consistent with typical rhGH response patterns reported in the literature. The relatively low rhGH dosage regimen used in this population was selected based on clinical considerations: lower doses may help minimize theoretical concerns regarding tumor recurrence, while still achieving effective growth stimulation. Furthermore, the slow progression of skeletal maturation—reflected by stable BA/CA ratios—suggests a prolonged growth period, which may contribute to improved final height outcomes.

GH therapy promotes linear growth velocity in CP patients, partly due to the growth hormone deficiency in this population. Growth rate was 7.45[6, 11.78], 7.9 ± 3.05 (n=17), and 7.17 ± 3.09 (n=11) cm per year during GH therapy with a decreasing growth rate. The growth rate was consistent with other studies (25–28). The dosage of GH in each year was similar. The choice of smaller GH dosage for CP was based on the following reasons: Firstly, lower GH dosage may theoretically minimize the risk of tumor recurrence (29). Secondly, it is shown that lower dosage of GH can effectively achieve a satisfactory growth rate. Lastly, the small progression in bone age in CP patients provided with an extended linear growth period (30, 31), thereby leading to an optimal final height possibly.

ISS patients demonstrate a growth velocity 9.33 ± 1.92, 8.45[7.35, 8.8], and 7.18 ± 0.96 (n=9) cm annually, exhibiting a decline in growth velocity by approximately 1 cm per year. Over the course of the treatment, the GH dosage augmented by 14%, but the IGF-1 SDS did not show a significant rise (P = 0.1331), implying growth hormone resistance during GH therapy (32). Increasing GH dosage to achieve optimal growth rate was also observed by other studies (33–35). The underlying mechanisms of GH resistance and optimal dosage increase in ISS patients may lie in the following aspects: (1) ISS patients may be in a state of partial GH and/or IGF-1 resistance, presenting with relatively short stature before GH therapy. Therefore, a higher dosage of GH is required for patients with ISS. (2) CP patients had a similar growth velocity with a stable GH dosage, indicating they maintain sensitivity to GH during the treatment period, while ISS patients do not. A general decline in GV and an increase in rhGH dosage in the ISS group suggest GH resistance. (3) IGF-1 SDS in ISS group decreased with no statistical significance, and the effect size suggests a negligible difference in the data. Thus, a higher dosage of GH is needed to bring the IGF-1 SDS up to the desired level, thereby promoting growth and development. This aligns with existing evidence that high-dose rhGH is recommended for optimal growth response in children with ISS (32).

The CP group exhibited a mean bone age delay of approximately 2 years, predominantly due to deficiency in gonadal and adrenal sex hormones. This is consistent with evidence that sex hormones contribute to skeletal maturation even before puberty (36, 37), and aligns with the prepubertal status (predominantly Tanner stage I) observed in these patients. As mentioned in the Table, the baseline age of CP group is significantly higher than that of ISS group, but the Tanner stages of the two groups are almost the same (P = 0.999), and they are basically in pre-puberty. For three years of GH therapy, the bone age progressed by about 1 and 2.7 years in two groups, because of profound absence of multiple hormones in CP patients. In our study, patients with GnRHa and AIs were excluded, because these factors may remarkably interfere bone maturation (16). Over a three-year period, bone age progressed in both groups, and ISS patients had more bone age development.

From baseline, the BA/CA ratio in the CP group was significantly lower than that in the ISS group, and this difference persisted throughout rhGH therapy, indicating delayed skeletal maturation in children with CP. Notably, bone age advanced more slowly than chronological age during treatment, which may have prolonged the window of linear growth. This extended growth period likely contributed substantially to the greater height gain and higher near-adult height observed in the CP group. Given that skeletal immaturity is a well-established determinant of growth potential, the observed differences in height outcomes may be largely attributable to maturational timing rather than intrinsic differences in GH sensitivity. While preserved GH responsiveness, consistent with the diagnosis of isolated GH deficiency in many CP patients, cannot be excluded as a contributing factor, the role of delayed bone age should be considered primary in interpreting these results (38).

IGF-1 level was regularly measured during therapy. The IGF-1 SDS in CP patients increased, despite the P-value not reaching statistical significance, the large effect size (Cohen’s d > 1) suggests a meaningful difference in the data. However, the change in IGF-1 SDS in ISS group was not statistically significant despite the ISS patients requiring nearly twice the dosage used for CP patients. This observation suggests a partial IGF-1 resistance in the ISS group, as they showed negligible improvement in IGF-1 SDS compared to the CP group, which responded well to standard hormone dosages. The differing responses to GH and IGF-1 between the two groups highlight the necessity for individualized GH dosing strategies in managing CP and ISS patients. Customizing hormone dosage based on these findings is crucial for optimizing treatment outcomes in different disease populations.

Despite these observations, several limitations should be acknowledged. First, the ISS patients was older at baseline, which may potentially have an unfavorable impact on evaluation of growth velocity.

Despite these observations, several limitations should be acknowledged. First, the study involved multiple outcome measures and repeated comparisons across several time points without adjustment for multiple testing, which may increase the risk of type I error—particularly given the limited sample size (n = 18 per group). Second, CP patients were older at baseline, which may affect growth velocity assessment. Third, detailed data on the exact timing of GH initiation relative to surgery were not available for systematic analysis, although therapy was initiated in accordance with consensus guidelines emphasizing stable disease remission. Additionally, detailed information on other pituitary hormone deficiencies in the CP group was not available for analysis, which limited our ability to assess the potential impact of complete endocrine status on growth outcomes. Future studies should collect comprehensive data on all pituitary axes. Although we adjusted for age and baseline height using ANCOVA, residual confounding from unmatched factors—particularly differences in skeletal maturation (evidenced by a lower BA/CA ratio in the CP group) and underlying growth potential—may have influenced the observed outcomes. Fourth, the mixed nature of missing data, at times due to attrition, at others due to variable clinical testing, may limit the robustness of longitudinal comparisons, particularly for secondary endpoints such as IGF-1 SDS and bone age progression. Plus, near-adult height in this study reflects height at last follow-up rather than confirmed adult height. Because children with CP typically exhibit delayed skeletal maturation compared to those with ISS, the observed height difference may partly reflect maturational timing rather than greater GH responsiveness. Taken together, these factors suggest that findings should be considered exploratory and interpreted as descriptive observations rather than evidence of intrinsic differences in GH sensitivity. Validation in larger, prospectively designed studies with confirmed adult height endpoints is warranted.

## 5 Conclusion

In this observational cohort, children with CP achieved comparable or greater improvements in growth velocity and final height than those with ISS, despite receiving a lower rhGH dose. The CP group maintained a significantly lower BA/CA ratio throughout treatment, indicating delayed skeletal maturation without acceleration. These findings likely reflect a combination of greater residual growth potential and intrinsic higher GH sensitivity.

## Data Availability

The datasets presented in this article are not readily available because The dataset contains patient information and cannot be made publicly available without informed consent. Requests to access the datasets should be directed to maojf@pumch.cn.
